# Evolutionary patterns of phosphorylated serines

**DOI:** 10.1186/1745-6150-6-8

**Published:** 2011-02-09

**Authors:** Yerbol Z Kurmangaliyev, Alexander Goland, Mikhail S Gelfand

**Affiliations:** 1 Institute for Information Transmission Problems (the Kharkevich Institute) RAS, Bolshoi Karetny pereulok 19, Moscow, 127994, Russia; 2 National Center for Biotechnology of the Republic of Kazakhstan, Valikhanov str., 13/1, Astana, 010000, Republic of Kazakhstan; 3 Faculty of Bioengineering and Bioinformatics, Moscow State University, Vorobievy Gory 1-73, Moscow, 119991, Russia

## Abstract

Posttranslationally modified amino acids are chemically distinct types of amino acids and in terms of evolution they might behave differently from their non-modified counterparts. In order to check this possibility, we reconstructed the evolutionary history of phosphorylated serines in several groups of organisms. Comparisons of substitution vectors have revealed some significant differences in the evolution of modified and corresponding non-modified amino acids. In particular, phosphoserines are more frequently substituted to aspartate and glutamate, compared to non-phosphorylated serines.

**Reviewers:**

This article was reviewed by Arcady Mushegian and Sandor Pongor.

## Findings

Post-translational modifications play an important role in diversifying protein structure and function [1,2]. Protein phosphorylation is one of the most important and widely distributed types of post-translational modifications. In eukaryotes, reversible protein phosphorylation plays a key role in the signal transduction and other processes [3,4]. Recent advances in mass spectrometry allowed for large-scale identification of phosphorylation events [5]. Analyses of these data have already revealed some specific structural and evolutionary features of phosphoserines. Phosphoserines tend to occur in intrinsically disordered regions [6-8] and regions corresponding to alternatively spliced gene segments [9]. Phosphorylated amino acids are more conserved than their non-phosphorylated counterparts [7,10-12]. Some very old phosphorylation events potentially can be common to organisms from *Archaea *to human [10].

Here we investigated another evolutionary aspect of protein modification sites. Since modified amino acids chemically are a distinct type of amino acids, in terms of evolution they might behave differently from their non-modified counterparts (on the top of the different level of conservation). To analyse differences in the evolution of standard amino acids and their modified counterparts, we reconstructed the evolution of phosphorylated amino acids in three groups of organisms. Particularly, we studied phosphorylation of serine in the human, fruit fly and yeast proteomes.

Phosphorylation sites were downloaded from the PHOSIDA [7] and PhosphoPEP [13] databases. For yeast and fruit fly we studied phosphoserines obtained in two high-throughput experiment each, by different groups of researchers [13-16]. For human we used datasets obtained in four different high-through experiments [17-20]. Phosphorylation is highly dynamic process, and the overlap of phosphorylation events identified in different experiments from various cell lines and tissues is relatively small. Sites observed to be phosphorylated in more than one high-througput experiment likely are modified in a more constitutive manner, or at least represent a more reliable dataset of phosphoserines.

We analysed the evolution of modification sites and their non-modified counterparts separately among eight vertebrates (human *Homo sapiens; *chimpanzee *Pan trogolodytes*; mouse *Mus musculus*; rat *Rattus norvegicus*; cow *Bos taurus*; dog *Canis lupus familiaris*; chicken *Gallus gallus*; and zebrafish *Danio rerio*), eleven fruit flies (*Drosophila melanogaster; D. yakuba*; *D. erecta; D. sechecellia; D. ananassae; D*. *pseudoobscura; D. persimilis; D. wilistoni; D. mojavensis; D. virilis; D. grimshawi*) and fifteen fungi (*Saccharomyces cerevisiae*; *S. paradoxus; S. mikatae; S. bayanus; Candida glabrata; S. castelli; Kluyveromyces waltii; K. lactis; Ashbya gossypii; Debaryomyces hansenii; C. albicans; Yarrowia lipolytica; Aspergillus nidulans; Neurospora crassa; Schizosaccharomyces pombe*). Orthologs of modified *H. sapiens *proteins were obtained from HomoloGene [21]; for *D. melanogaster*, from FlyBase [22]; and for *S. cerevisiae*, from FungalOrthogroups [23]. Only orthologs with the highest identity to the modified protein were selected from each species. Multiple alignments were constructed using ClustalW [24].

As mentioned above, the evolutionary features and frequencies of phosphoserines may depend on structural context. Especially, phosphoserines tend to occur within intrinsically disordered regions of proteins [6-8]. To take this into account, we analysed serines from disordered regions and ordered regions of phosphoproteins separately. Intrinsically disordered regions were predicted by PONDR VSL2 [25].

**Figure 1:**
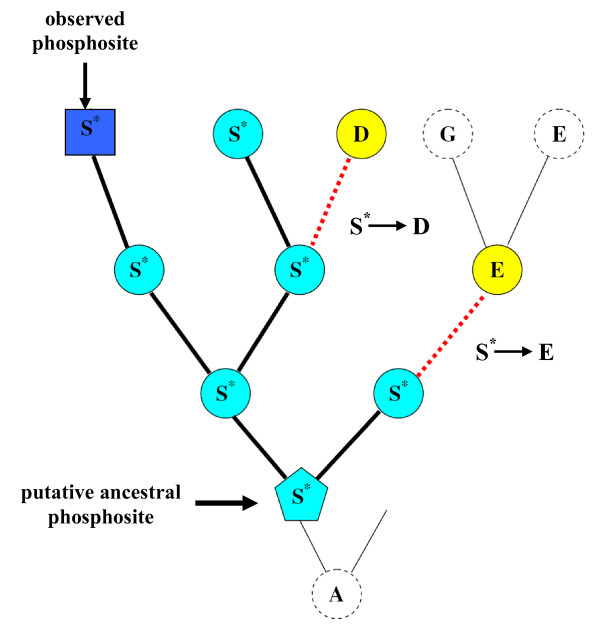
**Reconstruction of the evolution of phosphoserines**. The oldest serine residue (**light blue pentagon**) on the reconstructed tree is assumed to be phosphorylated, and the number of substitutions (**red dotted lines**) of putative phosphoserines (**light blue circles**) to other types of amino acids are counted.

For each phosphorylated serine, we have reconstructed the evolution of this site in the corresponding taxonomical group using a fast modification of the maximum likelihood algorithm (A. Goland, in preparation). Since we cannot reconstruct the moment in evolution when a residue had become modified, we assumed that it coincides with the oldest residue of the given type in a given tree (Figure 1). Then we calculated the number of substitutions of ancestral putative modification sites to other amino acids, and calculated the vectors of substitution frequencies.

Only a fraction of phosphoserines from the initial datasets were aligned to other types of amino acids in our data, and very small number of them occurred in ordered regions. Thus further analyses were performed only for serines from regions predicted to be intrinsically disordered. The final datasets of phosphorylated and non-phosphorylated serines included only sites that experienced at least one substituition to other types of amino acids and originated from disordered regions of phosphoproteins (Table 1). Some phosphorylation events were observed in more than one experiment, and this subset was also analyzed separately.

**Table 1:** Datasets of phosphorylated and non-phosphorylated serines

	*S. cerevisiae*	*D. melanogaster*	*H. sapiens*
*Initial sets of serine residues*			

all phosphoserines	7381	11785	11624

phosphoserines observed more than once	1649	3137	2589

non-phosphorylated serines	103682	202574	243968

*Serines with at least one substitution to other types of amino acids, within ordered regions*			

all phosphoserines	215	180	434

phosphoserines observed more than once	21	38	43

non-phosphorylated serines	20459	13826	26350

*Serines with at least one substitution to other types of amino acids, within disordered regions*			

all phosphoserines	3666	2482	4277

phosphoserines observed more than once	857	611	906

non-phosphorylated serines	31815	42424	78120

The number of serines in each set is given. Only serines from disordered regions, with at least one substitution to other types of amino acids, were analyzed (the last three lines).

The control sets consisted of non-modified serine residues from disordered regions of the same proteins. To measure the statistical significance of the difference between substitution vectors of modified and non-modified serines we performed bootstraping of control sets. To do that that, we generated 10000 random control sets of non-phosphorylated serines. Each control set was of the same size as the corresponding phosphorylated set (generic sets and subsets of reliable phophosites).

Structural features of phosphoserines may not be limited to disorder of surrounding protein regions, and may include other specific properties such as secondary structures, solvent availability etc. Therefore, to maximally eliminate the confounding effects, we created additional control sets containing non-modified serines located at the same protein regions as modification sites. Non-modified serines, was collected at the maximal distance of 10, 11 and 9 amino acid residues from phosphoserines, for yeast, fruit fly and human respectively. Again, the size of the control sets was the same as the size of the respective phosphoserine sets.

**Figure 2:**
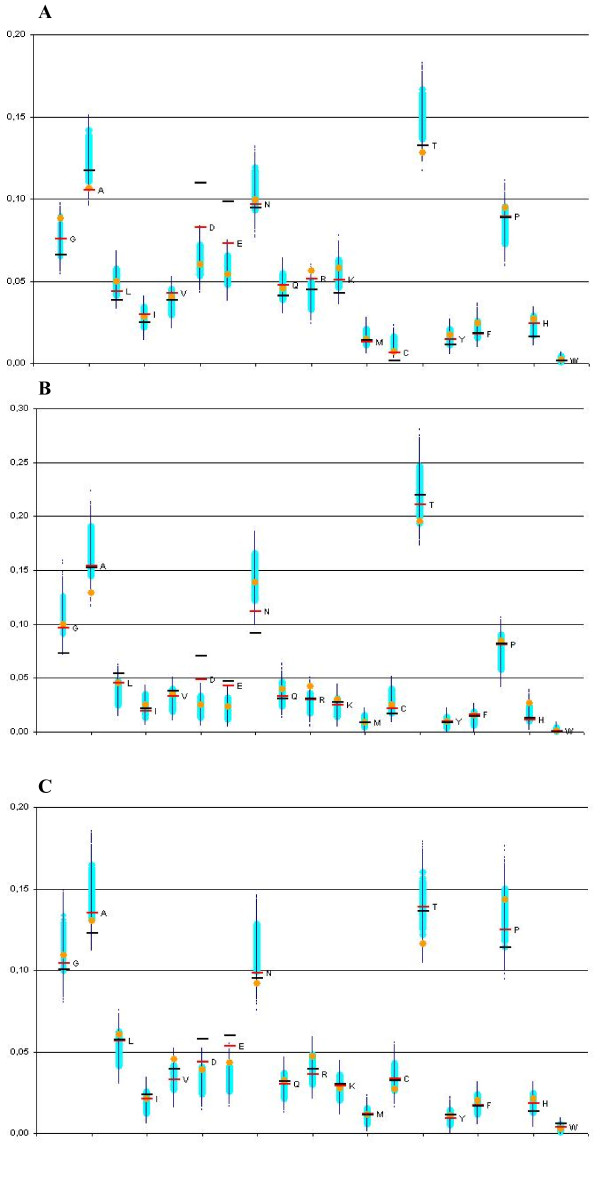
**Substitution vectors of serines to other types of amino acids**. Frequency of substitution of serines from disordered phosphoprotein regions among fungi (**A**), fruit flies (**B**) and vertebrates (**C**): for all phosphoserines - **red bars**; for phosphoserines observed in more than one experiment - **black bars**; 10000 control sets - **clouds of large light and dark small blue dots**, respectively; the additional control set of nearest non-phosphorylated serines - **orange dots**.

Differences in the substitution vectors between phosphorylated and non-phosphorylated serines from disordered regions varied among different groups of organism, but some trends were stable and significant (Figure 2). Rather unexpectedly, we did not observe any preference for substitution of phosphoserines to other aminoacids that may be phosphorylated, that is as threonine and tyrosine. At the same time, phosphorylation converts serine into a negatively charged amino acid, and, as one can see in Figure 2 in all three datasets phosphoserines are more frequently substituted to aspartate and glutamate than non-phosphorylated serines. In both cases the substitution rates of phosphoserines are much higher than in all bootstraps of control sets (P-value << 10^-4^). In the case of the more reliable subsets of phosphoserines observed in several experiments, the subtitution rate to aspartate and glutamate is even higher, and also lies outside the interval of bootstraps that in this case is wider, as the sample size is smaller. At that, artificial substitution of serine to aspartate and glutamate, called phosphomimetic mutation, is widely used to confirm phosphorylation of serine [26,27].

There are considerable other shifts of substitution rates common to all three taxa. Particularly, phosphoserines are relatively rarely substituted to alanines and cysteines (Figure 2). However, in these cases, the control-set substitution vectors of non-phosphorylated serines located in the same regions as phosphoserines were also shifted in the same direction as phosphoserines (as compared to all non-phosphorylated serines). Hence, these shifts are likely related not to modifications, but to specific features of these regions.

The rates of substitutions to aspartate and glutamate in the additional control sets of nearest non-phosphorylated serines also are not shifted, with the exception of vertebrates where they are also shifted toward higher values (but still to a much weaker extent than in case of phosphoserines). Note that these control sets may be contaminated by phosphoserines. Indeed, phosphoserines tends to co-occur, forming clusters [28]. Therefore the sets of nearest non-phosphorylated serines likely contain phosphoserines which were not detected yet. Removing these phosphoserines would increase the significance of our observations.

The comparison with nearest non-phosphorylated serines takes into account the fact that phosphoserines tend to occur in intrinsically disordered regions. Methods used in large-scale phosphoproteomic experiments are based on selection of negatively charged peptides which results in a bias towards enrichment of phosphopeptides with acidic residues [29,30]. This fact, coupled with the fact that phosphoserines may shift positions within rapidly evolving disordered regions [31] and general problems of alignments of such regions could distort our analysis. But this would have the same influence on our control sets of non-modified serines from the same regions of proteins. Hence the observed differences between these controls and phosphoserines cannot be explained by such artifacts.

In addition to serine phosphorylation, we analysed the evolution of another abundant type of protein modification, lysine acetylation. Recently two large datasets of human acetylation sites became available [32,33]. We observed some differences between substitution vectors of acetylated and non-acetylated lysines, but the results obtained for these two sets of acetyllysines were discordant (data not shown). As noted in one of these papers [32], the spectrum of acetylated proteins is different between these two datasets obtained from different tissues. We observed that less than 2% of sites are common for both datasets. It is seems that the available acetyllysine data are not sufficient for meaningful analysis.

It should be taken into account that our substitution vectors are probably enriched with false-positive phosphosites. This results from of our over-simplified assumption that a site is modified from the first appearance of the corresponding residue in the evolutionary record. Additionally, phosphoserines from large-scale experiments may be false-positive sites. There is evidence that many phosphorylation sites could be non-functional or non-specific, as sometimes functional targets of phosphorylation are not particular sites, but entire protein regions [31,34,35]. On the other hand, the control sets could contain not yet detected phosphoserines. These false positives and false negatives should blur the differences between the substitution vectors of modified and non-modified residues. Most likely, the real level of differences is higher than the one observed here.

## Competing interests

The authors declare that they have no competing interests.

## Authors' contributions

YK and MG conceived the study. YK compiled the data. AG developed algorithms. YK and MG performed calculations. YK and MG analyzed the results and wrote the paper. All authors have approved the final version.

## Reviewers' comments

### Reviewer's Report 1

Reviewer 1: Arcady Mushegian - Stowers Institute, Kansas City, USA

#### Reviewer's comment

The idea of comparing of evolutionary substitution patterns of modified and non-modified residues in proteins is good, and the approach proposed by the authors, i.e., to reconstruct, using an ML model, the point at which the target of modification first emerged and then to see what it mutates to, is probably the only computational approach plausible at the moment.

I trust the authors that their implementation of this approach is technically sound, but, unfortunately, this is hard to ascertain from the submitted version of the manuscript, which reads as a preliminary draft devoid of the quantitative details. This has to change - please provide at least the following:

1. The collection of phosphorylated and acetylated sites: how many sites of each type in each organism are there?

##### Author's Response

A table with a description of the final datasets used for the construction of substitution vectors has been added to the revised version (Table 1).

#### Reviewer's comment

2. The phosphorylation sites at least (also acetylated sites?) are said to occur more often in the intrinsically disorded regions. Taking the non-globular regions in the proteins (which can be identified, e.g., using Wootton and Federhen's SEG program) as a proxy for "intrinsic disorder", can it be said that the actual sample of modified residues that the authors were working with is indeed more commonly occurring in such regions? And how does this sit with the ability to align the proteins in these regions?

##### Author's Response

We predicted intrinsically disordered regions and recalculated substitution vectors separately for serine residues from disordered and ordered regions. Most of phosphoserines from the initial datasets came from protein regions predicted to be disordered (Table 1). Problems with alignments of such region are discussed in the revised version. Additional controls of non-modified serines from same regions of proteins were introduced to address this problem.

#### Reviewer's comment

3. The "control sets" of non-modified serines (more accurately, not-observed-to-be-modified serines): are these found in the disordered/non-globular regions to the same extent as the modified ones? If not, the controls may be biased with regard to amino acid composition and to the regions of the protein molecules (e.g., buried vs exposed) - test this directly please.

##### Author's Response

Indeed, the amino acid composition of disordered regions and regions with a regular structure differs strongly. As described in response to comment #2, in the revised version we considered both phosphosrylated and non-phosphorylated serines from disordered and regular regions separately. Moreover, as discussed in the revised text, sets including only closest non-modified serines provide an even better control for artifacts that could be caused by specifics of regions surrounding modification sites.

#### Reviewer's comment

4. The trends that the authors discuss are interesting but weak - to what extent this may be explained by the small sample sizes? What was the statistical test for which the P-values are reported?

##### Author's Response

The initial dataset of serines were large enough, but only a fraction of them were substituted to other amino acids as demonstrated by evolutionary reconstruction. The final datasets are described in Table 1.

To measure the statistical significance, we used bootstraps of control sets of non-modified serines. For all phosphoserines and, separately, for the subset of phosphoserines observed in more than one experiment, we generated 10000 random sets of non-modified serines of appropriate size. For additional controls using neighbouring sites, we compiled sets of nearest serines of the same size as the corresponding sets of phosphorylated serines.

#### Reviewer's comment

5. In vertebrates, the "neighboring" serines from control set 2 seem to be faithfully following the trend towards change into D or E, with some separation from the control set 1. If this trend withstands the possible correction proposed in #2, perhaps this means that, in a "disordered" region that has several serines, any or all of them may targets of phosphorylation. Perhaps then it would be interesting to sum the substitution vectors over the region that has several serines, at least one of which is phosphorylated (i.e., how likely is it that at least one serine in this region is substituted by amino acid X?)

##### Author's Response

The phosphoserines tends to cluster in the sequence [28]. Thus, as discussed in the revised version, the control set consisting of nearest non-phosphorylated serines could be contaminated by false-negative phosphoserines, not yet detected in experiments. On the other hand, as the trend in the control set of nearest serines is weaker, averaging of the substitution vectors would simply dilute the observation.

### Reviewer's Report 2

Reviewer 2: Sandor Pongor - International Centre for Genetic Engineering and Biotechnology, Trieste, Italy

#### Reviewer's comment

There is mounting evidence in recent years that the study of post-translational modifications has important lessons for understanding diverse aspects of protein evolution. It has been noted among others that phosphorylated sites tend to occur in those segments of the proteins that are intrinsically disordered and/or correspond to alternative splice sites. Currently there are insufficient data on the conservation of modified sites. Kurmangalyev and associates address this problem using carefully selected datasets and well-designed statistical analyses.

The authors conclude that there are significant differences in the evolution of modified and corresponding non-modified amino acids. In particular, phosphoserines are more frequently substituted to aspartate and glutamate, compared to non-phosphorylated serines. Similarly, acetyllysines are more rarely substituted to isoleucine and valine. These findings underline the importance of post-translational modifications when discussing the variation of residue conservations within sequence regions. The methodology is straightforward and sound and will be a useful template for future studies. The authors may want to add a few examples for situation where this approach can or can not be used.

##### Author's Response

As discussed in the revised text, the analysis of a newly available dataset of human acetylation sites [33] did not confirm our initial observations. This is likely due to low reproducibility of currently available datasets of avetyllysines (the overlap between two datasets is extremely small). This suggests that conclusions based on such analyses should be done carefully, on data obtained from different sources and for a variety of organisms. We have encountered a similar problem with phosphothreonines and phosphotyrosines, where the datasets were simply too small for reliable conclusions.
